# Overexpression of *AtEDT1/HDG11* in Chinese Kale (*Brassica oleracea* var. *alboglabra*) Enhances Drought and Osmotic Stress Tolerance

**DOI:** 10.3389/fpls.2016.01285

**Published:** 2016-08-30

**Authors:** Zhangsheng Zhu, Binmei Sun, Xiaoxia Xu, Hao Chen, Lifang Zou, Guoju Chen, Bihao Cao, Changming Chen, Jianjun Lei

**Affiliations:** ^1^College of Horticulture, South China Agricultural UniversityGuangzhou, China; ^2^National Engineering Research Center of Plant Space Breeding, South China Agricultural UniversityGuangzhou, China; ^3^Key Laboratory of Biology, Innovation and Utilization for Germplasm Resources in Horticultural Crops in Southern China, College of Horticulture, South China Agricultural UniversityGuangzhou, China

**Keywords:** *AtEDT1/HDG11*, drought, osmotic stress, abscisic acid, auxin, signaling response

## Abstract

Plants are constantly challenged by environmental stresses, including drought and high salinity. Improvement of drought and osmotic stress tolerance without yield decrease has been a great challenge in crop improvement. The *Arabidopsis ENHANCED DROUGHT TOLERANCE1/HOMEODOMAIN GLABROUS11* (*AtEDT1/HDG11*), a protein of the class IV HD-Zip family, has been demonstrated to significantly improve drought tolerance in *Arabidopsis*, rice, and pepper. Here, we report that *AtEDT1/HDG11* confers drought and osmotic stress tolerance in the Chinese kale. *AtEDT1/HDG11*-overexpression lines exhibit auxin-overproduction phenotypes, such as long hypocotyls, tall stems, more root hairs, and a larger root system architecture. Compared with the untransformed control, transgenic lines have significantly reduced stomatal density. In the leaves of transgenic Chinese kale plants, proline (Pro) content and reactive oxygen species-scavenging enzyme activity was significantly increased after drought and osmotic stress, particularly compared to wild kale. More importantly, *AtEDT1/HDG11*-overexpression leads to abscisic acid (ABA) hypersensitivity, resulting in ABA inhibitor germination and induced stomatal closure. Consistent with observed phenotypes, the expression levels of auxin, ABA, and stress-related genes were also altered under both normal and/or stress conditions. Further analysis showed that AtEDT1/HDG11, as a transcription factor, can target the auxin biosynthesis gene YUCC6 and ABA response genes ABI3 and ABI5. Collectively, our results provide a new insight into the role of *AtEDT1/HDG11* in enhancing abiotic stress resistance through auxin- and ABA-mediated signaling response in Chinese kale.

## Introduction

Drought and osmotic stress can seriously limit plant growth and productivity. Plants have developed multiple strategies to cope with drought and osmotic stress. These normally involve a mixture of stress avoidance and tolerance adaptations, which produce a range of changes at the morphological, physiological, cellular, and molecular levels (Shinozaki and Yamaguchi-Shinozaki, 2007; Ashraf, 2010). It is well known that the most efficient and important mechanisms for plants to cope with water deficit stress include maximization of water uptake through the development of deep and extensive root systems and/or minimization of water loss by stomatal closure and reduction of stomatal density (Yu et al., 2008, 2013).

Drought causes dehydration when root uptake of water from soil is insufficient to meet the transpirational requirements of plants (Jeong et al., 2010; Yoo et al., 2010). It has been reported that drought-resistant plants usually have deeper and more highly branched root systems than drought-sensitive plants. Ease of access to water and nutrients is a factor potentially limiting plant growth (Price and Tomos, 1997; Kondo et al., 2000; Yu et al., 2008; Uga et al., 2013). Literature has documented that overexpression of *AtCKX*, *AtNAC1*, *OsDRO1*, *OsNAC5*, and *OsNAC10* enhanced root depth and/or thickness and led to significantly improved drought tolerance (Xie et al., 2000; Jeong et al., 2010, 2013; Uga et al., 2013).

It is well known that the majority of plant transpiration occurs through stomatal pores (Yoo et al., 2010). Thus, water loss during transpiration in the stomata is a key determinant of drought tolerance (Yu et al., 2008). Plant transpiration rates can be modulated by stomatal density and/or stomatal movements (Masle et al., 2005; Yoo et al., 2010). A pair of specialized guard cells wield control over the size of stomatal apertures, which are influenced by many factors, such as illumination, water supply, CO_2_, abscisic acid (ABA), reactive oxygen species (ROS), and calcium and potassium ions (Berger and Altmann, 2000; Von Groll et al., 2002). Stomatal density is also variable and is set according to the environmental factors prevailing during leaf development. In addition to these exogenous factors, stomatal density is also subject to genetic control (Berger and Altmann, 2000; Masle et al., 2005; Shpak et al., 2005). Recently, a number of genes controlling stomatal development have been identified. It has been reported that overexpression of *AtERECTA*, *AtGTL1*, *AtSDD1*, and *OsSIK1* significantly enhanced drought tolerance in plants and was also associated with reduction of stomatal density (Masle et al., 2005; Ouyang et al., 2010; Yoo et al., 2010).

The homeodomain-leucine zipper (HD-Zip) superfamily of transcription factors is unique to the plant kingdom. They can be classified into four subfamilies, according to a set of distinctive features that include DNA-binding specificities, gene structures, additional common motifs and physiological functions (Ariel et al., 2007). Some HD-Zip proteins participate in organ and vascular development or meristem maintenance, while others mediate the action of hormones or are involved in responses to environmental conditions (Ariel et al., 2007; Agalou et al., 2008). In recent years, many efforts were undertaken to elucidate the functions of HD-Zip genes, and found a subset of genes is regulated by drought (Agalou et al., 2008). These include HD-Zip family I gene families *Athb-7* and *Athb-12* from Arabidopsis and *Oshox22* from rice (Söderman et al., 1996; Olsson et al., 2004; Ré et al., 2014; Zhang et al., 2012). The *Arabidopsis HOMEODOMAIN GLABROUS 11* (*HDG11*) encodes a protein of the class IV HD-Zip family, which has two known conserved domains, the homeobox domain and the START domain, and was first identified as a negative regulator of trichome branching development (Nakamura et al., 2006; Khosla et al., 2014). *AtEDT1/HDG11* was identified as improving drought tolerance in the *Arabidopsis enhanced drought tolerance1* (*edt1*) mutant through T-DNA inserted in the 5′-untranslated region of *HDG11*, resulting in the overexpression of HDG11, reduced stomatal density, and an improved root system (Yu et al., 2008). Additionally, ectopic expression of *AtEDT1/HDG11* has been demonstrated to significantly improve drought tolerance in rice, cotton, and pepper, without yield penalty, indicating that it is a promising candidate gene for crop stress tolerance development (Yu et al., 2013, 2016; Zhu et al., 2015). However, the underlying molecular mechanisms are still not well-studied.

In this work, *Arabidopsis Enhanced Drought Tolerance1/HOMEODOMAIN GLABROUS 11* (*AtEDT1/HDG11*) was observed to be overexpressed in Chinese kale. Our results demonstrate that the overexpression of *AtEDT1/HDG11* in Chinese kale is not only responsible for its enhanced drought and osmotic tolerance, but also significantly improves biomass under both normal and stress conditions. *AtEDT1/HDG11*-overexpression lines also alter the expression of ABA and auxin biosynthesis, and/or the expression of response genes. These findings may provide a new insight into the mechanism of abiotic stress tolerance in Chinese kale, and represent an important genetic engineering approach for the improvement of stress tolerance in crops.

## Materials and Methods

### Construction and Generation of Overexpression Plants

The full-length cDNA of *AtEDT1/HDG11* was isolated from the PCB2004-HDG11 plasmid vector (Yu et al., 2008) via the PCR specific primers AtHDG11-FOR and AtHDG11-REV (Supplementary Table 1). The sequence-confirmed fragment was cloned into pCAMBIA1301-Cry2Aa2-PIM. The construct was transformed into the Chinese kale (*Brassica oleracea* var. *alboglabra*) inbred line 25 using *Agrobacterium tumefaciens*-mediated transformation. The PCR and qRT-PCR analysis indicated that 12 T_0_ independent transgenic plants were generated; the Southern blot analysis indicated that most transgenic lines were harboring one copy. The positive T_2_ transformants were prescreened for drought resistance in the greenhouse and more than 80% of transgenic lines showed improved drought tolerance during the seedling stage. Several drought-tolerant lines (i.e., OE-1, OE-2, OE-3) were selected for further analysis (Supplementary Figure 1).

### Identification of Morphological Characterization of Transgenic Chinese Kale

For morphological characterization of plants, transgenic and wild-type (untransformed) plants were grown under standard or stress conditions. Leaves of the same age and same relative position were sampled from transgenic and wild-type plants during the seedling stage. The area was measured using the portable leaf area meter (CI-203 Handheld Laser Area Meter, USA). During the reproductive stage, the plant inflorescence, pedicel, siliques, and epidermal cells between the first and second leaf length were measured. Subsequently, siliques and seeds were counted.

### Morphological Characterization of Transgenic Plant Roots

For measurement of root elongation, the wild-type and transgenic seeds were germinated and then grown on an MS medium. The 7-day-old seedlings were used for hypocotyl length and root length measurement. To better observe the root system, transgenic and wild-type plants were grown in a nutrient solution. The number of roots was counted and the root length was measured. For measurement of roots in the soil, wild-type and transgenic seeds were transplanted to pots with the substrate (Tref BIO, Norway), and grown in a greenhouse under standard growth conditions for further research. For data analysis, the 8-week-old transgenic and wild-type plants were carefully removed from their pots. Subsequently, the soil was carefully separated without damaging the roots, and the root biomass was measured as fresh weight.

### Drought Tolerance Assay of *AtEDT1/HDG11* Transgenic Chinese Kale

For drought tolerance tests of plants in the seedling stage, 35-day-old wild-type and transgenic seedlings were subjected to drought treatment in greenhouse by withholding water for 5 days (severe drought stress), re-watering for recovery for 4 days and then calculating the survival rate of seedlings. To evaluate the maximum photochemical efficiency of PSII under moderate drought stress (water withheld for three days), *F*v/*F*m values were measured by the Opti-Science OS-30p (Opti-Sciences, USA). After treatment with drought stress plants leaves were harvested and frozen in liquid nitrogen for a chlorophyll, proline and H_2_O_2_ contents and superoxide dismutase (SOD) activity assay.

To measure stomatal aperture, 35-day-old seedlings underwent a 2-day drought treatment, and the imprint method was then used for stomatal measurement as previously described (Zhu et al., 2015). To measure water loss rates, roots were detached from the transgenic and wild-type plants, respectively. Plant shoots were weighed immediately after detachment, placed on a plate on a laboratory bench, and were then weighed at designated time intervals (0, 0.8, 1.6, 2.4, 3.2, 4.0, 4.8, 5.6, 6.4, and 7.2 h). The proportion of fresh weight loss was expressed using the initial weight of the plants. For data analysis, 10 plants were used for treatment.

To test Chinese kale drought tolerance at the flowering stage, the transgenic and wild-type plants were subjected to a water deficit of withheld watering for 3–5 days (3 days for the early stage and 5 days for the late stage). The plants were re-watered while drought stress symptoms (e.g., leaf wilting) were clearly observable in the transgenic lines, and then plants were left to recover for 15 days. Repeated drought treatment was sustained until the siliques and seeds were obtained and counted.

### Osmotic Tolerance Assay of *AtEDT1/HDG11* Transgenic Chinese Kale

For the osmotic tolerance (PEG and salt) test at the seedling stage, 40-day-old transgenic and wild-type plants underwent treatment with 25% PEG and 250mM NaCl, respectively. Osmotic tolerance treatment was sustained for 30 days and after a re-watering recovery for 7 days, the survival rate was calculated and the biomass of surviving plants was weighted. For further analysis of osmotic tolerance, transgenic plants were subjected to an osmotic stress treatment for 10 days, and leaves of similar developmental stages from stress-treated plants or normal control plants were sampled for proline and H_2_O_2_ contents and SOD activity measurement.

### Quantification of Free IAA Contents

Free IAA from the indicated tissues was extracted as described by Pan et al. (2010). Free IAA contents were measured using a Phytodetek^TM^ IAA Test kit (Agdia, Arizona, CA, USA) according to the manufacturer’s instructions.

### Chlorophyll Content Measurement

To measure the chlorophyll content of transgenic plants and wild-type plants after exposure to 3 days of drought treatments, a 0.5 cm^2^ disk was cut from the middle of the leaf blade, and chlorophyll content was calculated and expressed as mg/g FW (Loukehaich et al., 2012).

### Measurement of Proline Content

Leaves of similar developmental stages from stress-treated plants or normal control plants were used for proline content measurement. Proline was assayed as described by Bates et al. (1973).

### Determination of SOD Activity

For measurement of the SOD activities, 0.1 g of leaves was sampled and homogenized in an ice-cold mortar using a 50 mM sodium phosphate buffer (pH 7.8) containing 1 % polyvinylpyrrolidone and 10 mmol L^-1^β-mercaptoethanol. Subsequently, centrifuged at 12,000 × *g* for 15 min at 4°C, the supernatant was used for the determination of SOD. Total SOD activity was measured by the SOD assay kit according to the manufacturer’s instructions (Nanjing Jiancheng Bioengineering Institute).

### Measurement of H_2_O_2_ Content

Frozen tissues were ground in liquid nitrogen. Ground tissues (50 mg) were soaked in a hydrogen peroxide assay kit lysis buffer (Beyotime). The extracts were clarified by centrifugation at 1,2000 × *g* for 15 min at 4°C. H_2_O_2_ concentration using the hydrogen peroxide assay kit according to the manufacturer’s instructions.

### Seeds Germination Test

For the germination assays, more than 40 seeds were placed on one MS agar medium containing different concentrations of ABA. To break the dormancy, seeds were incubated at 4°C for 2 days in the dark before germination and were subsequently grown in a growth chamber at room temperature. Seed germination was followed for 7 days. Seeds were counted as germinated when the radicles had emerged by 1 mm. The germination rate was calculated as a percentage of the total number of seeds planted. For data analysis the experiment was repeated at least three times.

### Stomatal Density and Aperture Measurements

To measure the stomatal density, fully expanded leaves in close proximity to one another were detached. Subsequently, the leaf surface imprint method was used, as previously described, to evaluate cells and stomatal density (Zhu et al., 2015). For statistical analysis of stomatal density, proximate leaves were sampled, and five plants were sampled for the wild-type and transgenic plants, respectively.

Fully expanded leaves of the same relative position and age were detached from the transgenic and the wild-type plants. The leaves were then placed in solutions containing 20 mM KCl, 5 mM MES-KOH (pH 6.15), and 1 mM CaCl_2_ and exposed to light at a photon fluency rate of 150 μM m^-2^sec^-1^ for 2 h for the stomata to fully open. Subsequently, ABA was added to the solution at 1–100 μM to assay the stomata closing. The inner edges of guard cells, the height of which was between 16 and 26 μm, were focused on by an optical microscope (Carl Zeiss; Germany), and the apertures of usually 30 to 50 stomata were measured.

### Measurements of Photosynthetic Rate, Transpiration Rate, and Water Use Efficiency

Photosynthesis (P) and transpiration (T) rates of transgenic and wild-type seedlings were measured using the portable photosynthesis system Li-6400XT (LI-COR, USA). This was done in the morning (9 to 11 AM), on the same plants mentioned above, before stomata observation. All of the photosynthetic measurements were taken at a constant air flow rate of 500 μmol s^-1^. The concentration of CO_2_ was 400 μmol mol^-1^ and delivered using the system’s CO_2_ injector (Li-Cor 6400-01). The chamber temperature was maintained at 26 ± 2°C, and the photosynthetic photon flux density at 1,200 μmol m^-2^ s^-1^. Three measurements were made for each plant, and five plants were used for both the wild-type and the transgenic plants. WUE was defined as *P*/*T* ratio and were derived from the measured *P* and *T*.

### Real-Time Reverse Transcription (RT)-PCR Analysis

Total RNA was prepared from tissues indicated in the figures by the TRIzol (Life, USA), and 1 μg of RNA from each sample was used for the reverse transcription reaction by the Prime Script^TM^ RT reagent kit with gDNA eraser (Takara, Japan). Quantitative real-time PCR analysis was performed on a Light Cycler 480 Real-Time PCR System according to the manufacturer’s instructions (Roche, Switzerland). The qPCR program included an initial denaturation step at 94°C for 8 min, followed by 40 cycles of 10 s at 94°C, 15 s at 56°C, and 30 s at 72°C. As an internal control, the *tubulin8* transcript was used to quantify the relative transcript level of each target gene in each sample. The gene accession numbers and sequences of all primers used for qPCR analysis are as described (Supplementary Table 1). The values represent the mean of three biological replicates.

### Promoter Analysis of Stress-Responsive and Phenotype-Related Genes

Promoter sequences at about 2 kb length upstream of the ATG start codon were obtained from BRAD (Cheng et al., 2011) with specific primers (Supplementary Table 1), and *cis*-elements in promoters found in the PLACE^1^ and Plant-CARE databases^2^.

### Transient Transactivation Assay

Promoters of four genes *ABI3*, *ABI5*, *EXPA5*, and *YUCCA6* were isolated from Chinese kale and cloned into the binary vector pEGFP. The effector construct 35S-*AtEDT1/HDG11* was also used in the transformation. An *AtEDT1/HDG11* N-terminal deletion without the HD domain was constructed in the same vector pEGFP as for the effector construct and used as a negative control. These reporter constructs were co-transformed into onion cells with either the 35S-*AtEDT1/HDG11* construct as effector or a modified 35S-*AtEDT1/HDG11* N-terminal deletion without the HD domain as a negative effector control (Yu et al., 2008). The transformed onion cells were observed with a confocal microscope (Carl Zeiss; Germany) using an excitation wavelength of 488 nm. GFP fluorescence intensity was quantified using ImageJ (Abràmoff et al., 2004).

### Statistical Analysis

Statistically significant differences (*P* < 0.05 or *P* < 0.01) were computed based on the Student’s *t*-tests. Data are the means ± SD of least three independent repeat experiments.

## Results

### Morphological Characterization of Transgenic Chinese Kale

**FIGURE 1:**
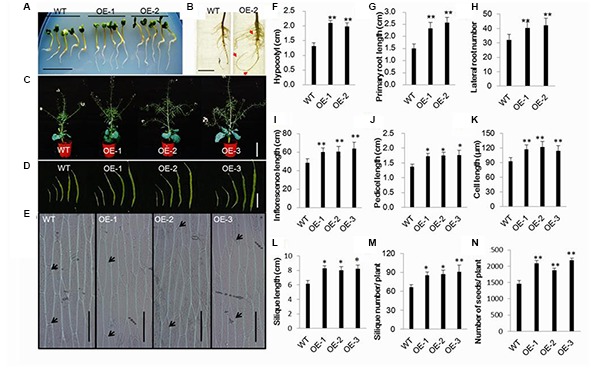
**Morphological characterization of transgenic Chinese kale plants. (A)** The primary root and hypocotyl of 7-day-old transgenic seedlings was longer than that of the wild-type (untransformed, WT) seedlings of the same age on the MS medium. Bar = 1.5 cm. **(B)** Root systems of 3-week-old transgenic and wild-type plants grown in a nutrient solution. Bar = 3cm. **(C)** Wild-type and transgenic plants in the flower stage. Bar = 30cm. **(D)** Transgenic and wild-type plants siliques 6, 10, 20, 30, and 50 day post-anthesis. Bar = 4cm. **(E)** Epidermal cells of the stems between the first and second leaf. **(F,G)** Hypocotyl and primary root length of 7-day-old seedlings. Values are mean ± SD (*n* = 10 plants). **(H)** Number of roots of 3-week-old transgenic and wild-type plants Values are mean ± SD (*n* = 10 plants). **(I**–**L)** Inflorescence, pedicel, epidermal cell and silique lengths of transgenic and wild-type plants. Values are mean ± SD. For analysis of inflorescence length 10 plants were used, respectively. Fifty-day-old siliques were used for silique and pedicel length measurement. Five siliques from the base of the main inflorescence of 10 plants. Epidermal cells of the stems between the first and second cauline leaves were examined. Measurements of five stems, 10 epidermal cells from each stem. **(M)** Silique number of transgenic and wild-type plants. Values are mean ± SD (*n* = 10 plants). **(N)** Seed numbers in transgenic and wild-type plants. Values are mean ± SD (*n* = 10 plants). *^∗^P* < 0.05 and *^∗∗^P* < 0.01 asterisks indicate Student’s *t*-test significant differences.

It this study, we generated *AtEDT1/HDG11*-overexpressing Chinese kale plants and the phenotype of Chinese kale was characterized at different developmental stages. During the seedling stage the transgenic plants showed auxin-overproduction phenotypes such as hypocotyl and primary root length, root hairs, and lateral numbers were significantly increased (**Figures 1A**,**B**,**F**–**H**; Supplementary Figure 2). A larger root system was also observed in transgenic plants during late periods of vegetative development (Supplementary Figures 3A,B). As a consequence, the fresh weight of 8-week-old Chinese kale was significantly improved in *AtEDT1/HDG11*-overexpressing lines (Supplementary Figure 3C). However, the root-to-shoot biomass ratio was almost unaltered, owing to the increased root biomass, which was nearly equivalent to the shoot biomass (data not shown).

During the reproductive phase, the plant height and main inflorescence length of transgenic lines were significantly improved in comparison to the wild-type plants (**Figures 1C,I**), and the increased elongation was mainly caused by increased cell length and width (**Figures 1E,K**; Supplementary Figure 4). Accordingly, transgenic plants had more siliques than wild-type plants (**Figure 1M**). In addition, compared to the wild-type control, pedicel and silique lengths were also significantly improved in transgenic plants (**Figures 1J,L**). The siliques are blunt in the wild-type plants compared to the transgenic lines (Supplementary Figure 5). Furthermore, the number of seeds for transgenic lines was higher than in the wild-type plant (**Figure 1N**).

### *AtEDT1/HDG11* Enhanced Drought Tolerance of Transgenic Chinese Kale

**FIGURE 2:**
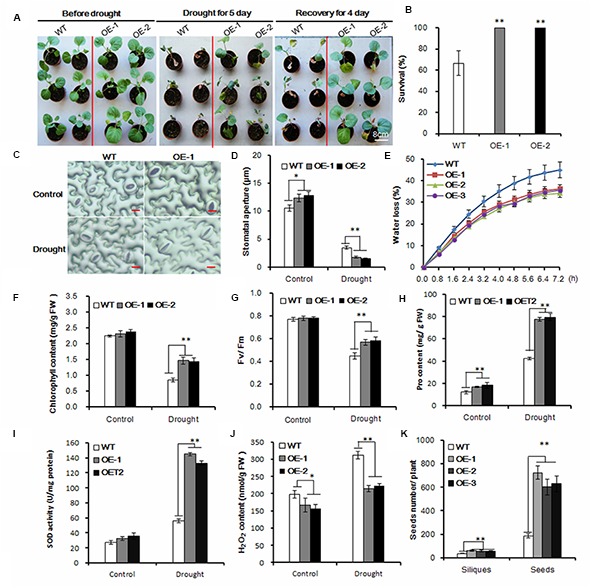
**Enhanced drought tolerance in transgenic Chinese kale. (A)** Drought treatment of 35-day-old transgenic plants in a greenhouse for 5 days and then recovery for 5 days. Bar = 8 cm. **(B)** Survival rate after 5-day drought treatment and 4 days of recovery. Values are mean ± SD (*n* = 50, *^∗∗^P* < 0.01). **(C)** Stomatal observation of the transgenic Chinese kale lines. Thirty-five-day-old plants were treated with drought stress for 2 days. The tests were repeated three times, and typical results are presented. **(D)** Stomatal apertures of the transgenic Chinese kale lines as shown in **(C)**. Values are mean ± SD (*n* = 50 stomata). **(E)** Comparison of water loss rate between transgenic and wild-type plants. Water loss was measured at the indicated time points and expressed as the percentage of the initial fresh weight (FW). Values are mean ± SD (*n* = 10 plants). **(F–J)** Chlorophyll content, chlorophyll fluorescence (*F*v/*F*m), Pro content, SOD activity, H_2_O_2_ content of transgenic and wild-type plants after 3-day water deficit treatment. Data represents mean ± SD (*^∗^p <* 0.05, *^∗∗^p* < 0.01). **(K)** Siliques and seeds of the transgenic and wild-type plants under drought stress conditions. Values are mean ± SD (*n* = 10 plants). Asterisks indicate Student’s *t*-test significant differences

Drought tolerance during the seedling stage is important for Chinese kale biomass establishment, so we carefully tested the drought tolerance in transgenic lines at the seedling stage. Under drought stress conditions, leaf rolling was significantly delayed in transgenic plants compared to wild-type plants (**Figure 2A**; Supplementary Figure 1). After 5 days of drought treatment and subsequent recovery for 4 days, 100% of the transgenic plants survived, when only 40% of the wild-type plants survived (**Figure 2B**). The majority of plant transpiration occurs via the stomatal pores, so we observed the stomata under drought conditions. After drought stress, the stomatal apertures of transgenic lines were smaller than those of the wild-type control (**Figures 2C,D**). Accordingly, 76% (46 of 60) of the wild-type stomata were open in contrast to only 28.3% (17 of 60) and 35% (21 of 60) in the transgenic lines OE-1 and OE-2, respectively. As a result, water loss in the transgenic plants was much slower than in the wild-type (**Figure 2E**).

To investigate the drought tolerance of transgenic Chinese kale, the chlorophyll content was measured. The chlorophyll content was about 72 and 69% higher in the transgenic lines OE-1 and OE-2, respectively, than in the wild-type plants under drought conditions (**Figure 2F**). Accordingly, the *F*v/*F*m levels were significantly higher in transgenic plants than in the wild-type plants under drought conditions (**Figure 2G**). Meanwhile, proline (Pro), a common compatible osmolyte in higher plants, was measured before and after 3 days of drought stress. The Pro content of the transgenic plants was higher than that of the control under normal conditions, and under drought stress increased more significantly in the transgenic plants compared with the wild-type controls (**Figure 2H**). Moreover, the activity of ROS-scavenging enzymes, such as SOD, was significantly increased in the *AtHDG11* transgenic plants compared with wild-type plants after stress treatments (**Figure 2I**), consistent with low H_2_O_2_ level detected in transgenic lines (**Figure 2J**). Therefore, our data indicate that the transgenic plants are better protected from oxidative damage during drought and salt stress.

Furthermore, we compared the drought tolerance performance of transgenic and the wild-type Chinese kale at the reproductive stage. Compared to the untransformed plants, the *AtEDT1/HDG11*-overexpressing lines have more siliques and seeds (**Figure 2K**). These results demonstrate that AtED1/HDG11 can significantly increase the drought tolerance of Chinese kale plants, likely at different developmental stages.

### Osmotic Tolerance Was Improved in *AtEDT1/HDG11*-Overxpressing Chinese Kale

**FIGURE 3:**
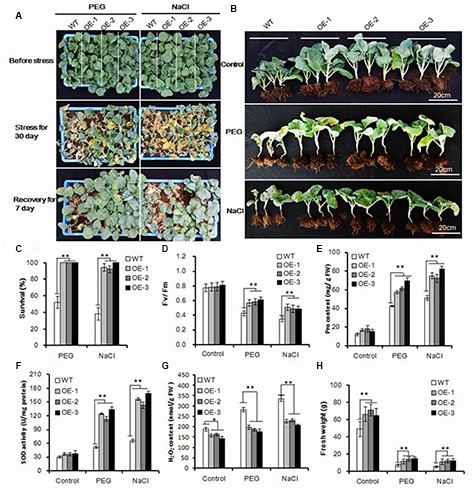
**Enhanced osmotic and salt tolerance in transgenic Chinese kale. (A)** Forty-day-old transgenic and wild-type plant treatment with 25% PEG 6000 and 250 mM NaCl for 30 days and recovery of 7 days. **(B)** Transgenic plants with or without stress treatment after recovery for 7 days. **(C)** Survival rate after 30-day stress treatment and 7 days of recovery. Values are mean ± SD (*n* = 50, *^∗∗^P* < 0.01). **(D–G)** Chlorophyll fluorescence (*F*v/*F*m), Pro content, SOD activity, and H_2_O_2_ content of transgenic plants subjected to stress treatment for 10 days. Data represents mean ± SD of three independent experiments (*^∗^p <* 0.05, *^∗∗^p* < 0.01). **(H)** Fresh weight of transgenic and wild-type plants with or without stress treatment. Values are mean ± SD (*n* = 10 plants, *^∗∗^p* < 0.01). Asterisks indicate Student’s *t*-test significant differences.

Drought stress was usually accompanied by high osmotic stress. To test whether the *AtEDT1/HDG11*-overexpressing plants were more osmotic-tolerant, we carried out PEG and salt tolerance tests in the greenhouse. For osmotic stress treatment, the 40-day-old seedlings were irrigated with 500 ML 25% PEG6000 or 250 mM NaCl solution for each pot every 3 days, respectively. After treatment with PEG6000 or NaCl for 30 days, the wild-type plants withered with many leaves dropping and dying, indicating severe osmotic stress in the plants. However, the *AtEDT1/HDG11*-overexpressing plants under the same conditions still showed relatively normal growth phenotypes (**Figures 3A,B**). After watering for recovery of 7 days, the survival rate of transgenic plants was the highest among all the plants treated with PEG6000 and NaCl (**Figure 3C**), respectively. Moreover, the *F*v/*F*m levels were significantly higher in transgenic plants than in the wild-type plants under osmotic conditions (**Figure 3D**). Similar to the results of drought stress, after treatment with PEG and NaCl transformed lines also showed higher levels of Pro content and SOD activity (**Figures 3E,F**), while H_2_O_2_ content was decreased (**Figure 3G**).

More importantly, the transgenic Chinese kale showed significantly improved fresh weight both under normal and osmotic stress conditions, (**Figure 3H**). Compared with normal conditions, the biomass of HDG11 overexpressing lines decreased approximately 80%, while 85% decreased in wild-type lines. Taken together, our results show that the *AtEDT1/HDG11*-overexpressing Chinese kale has significantly improved osmotic tolerance.

### Enlarged Leaf Area and Stomatal Size with Reduced Stomatal Density but Increased Water Use Efficiency in Transgenic Chinese Kale

**FIGURE 4:**
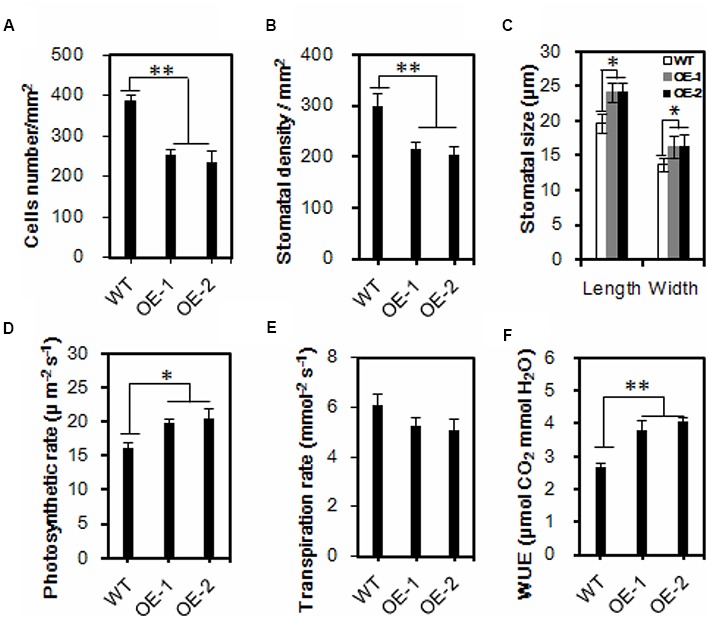
**Reduced leaf stomatal density, enlarged stomatal size and increased water use efficiency in the transgenic Chinese kale. (A,B)** Comparisons of cells and stomatal density. Three leaves were sampled for each plant, and five plants were sampled for both the control and the transgenic lines. Values are mean ± SD (*^∗∗^p* < 0.01). **(C)** Comparisons of stomatal dimension in transgenic and wild-type plants. Values are mean ± SD (*n* = 50, *^∗∗^p* < 0.01). **(D–F)** Comparisons of photosynthesis rate **(D)**, transpiration rate **(E)**, and WUE **(F)** in wild-type and transgenic Chinese kale plants. Values are mean ± SD (^∗^*P* < 0.05, ^∗∗^*P* < 0.01). Asterisks indicate Student’s *t*-test significant differences.

Under normal conditions, transgenic plants have exhibited a larger leaf area (Supplementary Figures 6A–C). It is well known that the majority of plant transpiration occurs through stomatal pores, and plant transpiration rates can be modulated by stomatal density and/or stomatal movements. Because the water loss was significantly delayed in transgenic plants, and to uncover the underlying mechanism, the leaf surface imprint method was used to evaluate the formation of cells and stomata (Supplementary Figure 7). The average cell density of the transgenic lines OE-1 and OE-2 was reduced by 34.7 and 39.2% compared with that of the wild-type, respectively (**Figure 4A**). Accordingly, the stomatal density was reduced by 27.9 and 39.9%, respectively (**Figure 4B**). While the stomatal density was decreased, the stomatal size was increased in the *AtEDT1/HDG11*-overexpressing lines (**Figure 4C**; Supplementary Figure 7). Compared with wild-type controls the average stomatal length in transgenic lines OE-1 and OE-2 was increased by 24.5 and 21.4%, respectively (**Figure 4C**). Correspondingly, the average stomatal widths of transgenic lines OE-1 and OE-2 were increased by 18.2 and 19.7%, respectively (**Figure 4C**).

Reduced stomatal density is known to affect water and CO_2_ exchange. We thus measured photosynthesis and transpiration rates of both the wild-type and the *AtEDT1/HDG11*-overexpressing Chinese kale plants at the seedling stage. Interestingly, the photosynthesis rate of transgenic line OE-1 and OE-2 was increased 22.5 and 26.8%, respectively, compared to the wild-type control (**Figure 4D**). Nevertheless, the transpiration rate in transgenic lines is lower than in wild-type plants (**Figure 4E**). Consequently, the water use efficiency (WUE) was significantly higher than in the wild-type control (**Figure 4F**).

### Transgenic Chinese Kale Was Hypersensitive to ABA

**FIGURE 5:**
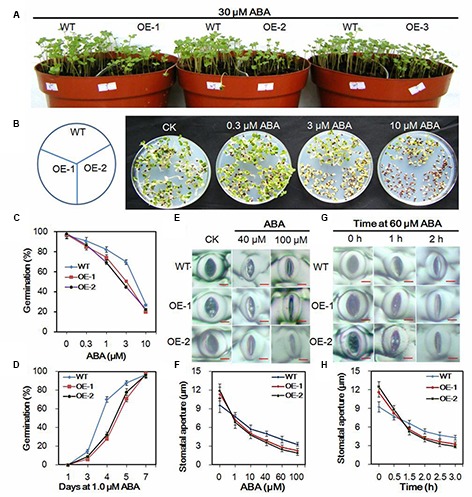
**Responses of transgenic and wild-type Chinese kale plants to ABA. (A)** Transgenic and wild-type seedlings treated with 30 μM ABA. **(B)** Seeds of Chinese kale sowed on MS agar plates with designated concentrations of ABA for 5 days. **(C)** Germination rate of Chinese kale with different concentrations of ABA for 5 days. **(D)** Germination in the presence of 1.0 μM ABA. **(E)** ABA-induced stomatal closing in transgenic and wild-type plants. Bar = 5 μm. **(F)** Stomatal apertures as a function of ABA concentration. Values are mean ± SD (*n* = 30–50 stomata per data point). **(G)** ABA (60 μM)-induced stomatal closing. Bar = 5 μm. **(H)** Stomatal aperture in 60 μM ABA at indicated time point. Values are mean ± SD (*n* = 30–50 stomata per data point).

During the seedling stage, the *AtEDT1/HDG11*-overexpressing lines showed higher sensitivity to ABA (**Figure 5A**). A test was performed to investigate the response of transgenic and wild-type plants to ABA. Without ABA, the germination rate of transgenic plants did not show an apparent difference from that in wild-type plants (data not shown). However, the transgenic plants demonstrated a dramatically delayed and reduced level of germination in the medium containing ABA, which indicated hypersensitivity of the transgenic plants to ABA (**Figures 5B–D**). After treatment with 3 μM ABA, only 50.4% of lines OE-1 and 44.7% of lines OE-2 germinated compared to 69.6% of the wild-type. Moreover, under the 1.0 μM ABA treatment, 70% of the wild-type germinated at day 4 compared to only 28.2 and 32.5% of lines OE-1 and OE-2, respectively.

Stomatal dynamic variation is a crucial ABA-regulated process. To investigate the ABA induced stomatal closure, experiments were performed at the concentration of 1–100 μM ABA to assay the stomatal closing. Under normal conditions, the stomatal apertures in transgenic plants were larger than in the wild-type plants (**Figure 5E**). However, after treatment with indicated ABA for 2 h, the guard cells were dramatically changed, and the stomatal apertures were smaller in transgenic lines than those in the wild-type plants (**Figures 5E,F**). Accordingly, when treated with 60 μM ABA for more than 1 h, the stomatal apertures in transgenic were smaller than in the wild-type plants (**Figures 5G,H**). Results further indicated that the stomatal closure in the transgenic plants was faster than in the wild-type under the ABA treatment conditions (**Figures 5E–H**).

### Expression Analysis of Phenotype- and the Drought Stress-Related Genes

**FIGURE 6:**
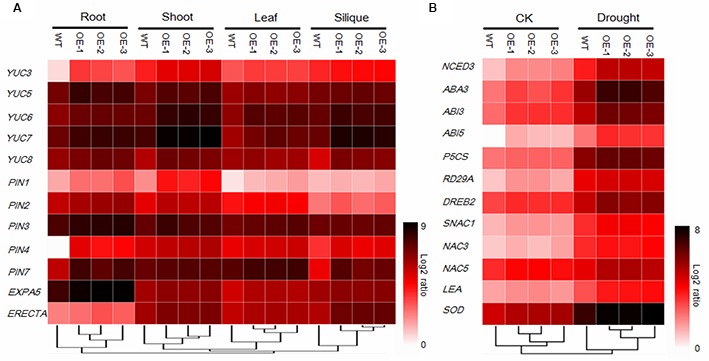
**Gene expression analysis of 12 development-related genes (A) and 12 stress-related genes under normal (CK) and drought stress conditions (B) using 384-well block high-throughput qRT-PCR.** Relative transcript levels after normalization to reference gene *tubulin8*. Values are the mean of three independent experiments and expressed as log2 ratios relative to expression in WT, results shown as a heat map. Gene expression profiles were organized by hierarchical clustering.

To uncover phenotype-related molecular mechanisms, the expression levels of 12 phenotype-related genes were investigated. *YUC3*, *YUC5*, *YUC6*, *YUC7*, and *YUC8* belong to the *YUC* gene family, which encodes the flavin monooxygenases proteins, which play essential roles in auxin biosynthesis and plant development (Cheng et al., 2006), were found to be significantly upregulated in the *AtEDT1/HDG11*-overexpressing plants (**Figure 6A**). Moreover, the *PIN1*, *PIN2*, *PIN3*, *PIN4*, and *PIN7* encoding auxin eﬄux facilitator proteins, which control auxin distribution and regulate cell division and cell expansion (Blilou et al., 2005), were increased. In addition, *EXPA5*, the cell-wall-loosening protein gene which controls root elongation (Xu et al., 2014) was also significantly upregulated in the transgenic plants (**Figure 6A**). *ERECTA*, which encodes a signaling molecule, and is a member of the family of the cell surface receptor kinase, has been demonstrated to enhance vegetative and reproductive organ development and stomata behavior (Torii et al., 1996; Yokoyama et al., 1998; Douglas et al., 2002; Shpak et al., 2004; Masle et al., 2005; Woodward et al., 2005; Bemis et al., 2013). Thus, up-regulation of auxin biosynthesis and response genes may influence the development in transgenic Chinese kale (**Figure 6A**).

To better understand the mechanisms of drought and osmotic tolerance in the *AtEDT1/HDG11*-overexpressing plants, the expression levels of 12 stress-related genes were also investigated. As shown in **Figure 6B**, the expression levels of *NCED3* and *LOS5/ABA3*, encoding the key enzymes in the ABA synthesis pathway, were up-regulated in the transgenic plants under both normal and drought stress conditions. *RD29A*, a stress responsive marker gene, was up-regulated. *P5CS*, encoding a key enzyme in the proline biosynthesis, *SOD*, encoding the key enzyme in Cu/Zn-SOD synthesis, and *LEA*, encoding the late embryogenesis abundant (LEA) protein, were strongly induced in the transgenic plants under drought stress. *DREB2*, encoding the dehydration responsive element binding protein, which induces a set of abiotic stress-related genes and imparts stress endurance to plants, was also up-regulated in transgenic plants under both normal and drought conditions compared to the corresponding wild-type. Moreover, *ABA-Insensitive3* (*ABI3*) and *ABA-Insensitive5* (*ABI5*), which encode proteins for ABA signaling response, were up-regulated in the transgenic plants. In addition, *SNAC1*, *NAC3*, and *NAC5*, which belong to the NAC family of transcription factors and have been well studied in response to drought and/or salt stress, were up-regulated under both the normal and stress conditions.

### *AtEDT1/HDG11* Can Target the Promoter of *ABI3*, *ABI5*, *EXPA5*, and *YUC6*

**FIGURE 7:**
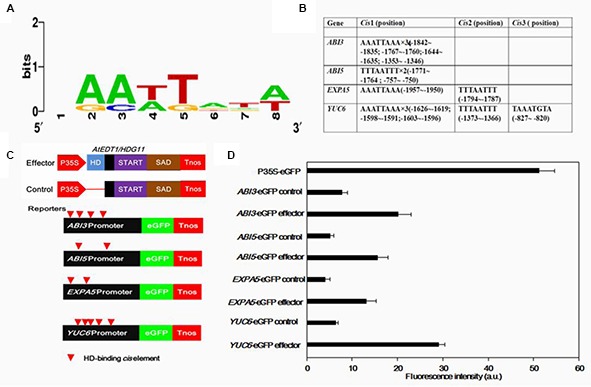
**Transient assays for the transactivation of *ABI3*, *ABI5*, *EXPA5*, and *YUC6* promoters by *AtEDT1/HDG11* in onion epidermal cells. (A)** Conservation logo of the HD-binding cis-element (L1-box). **(B)** Analysis of HD-binding *cis*-elements in promoters of *ABI3*, *ABI5*, *EXPA5* and *YUC6*. **(C)** Effector containing 35S-*AtEDT1/HDG11* or a modified 35S-*AtEDT1/HDG11* without the HD domain deleted server as a negative control. Enhanced green fluorescent protein (eGFP) reporter constructs containing the *ABI3*, *ABI5*, *EXPA5*, and *YUC6* promoter, respectively. The effector and reporter were transiently transformed into the onion. The 35S-eGFP construct served as a positive control. After incubation for 24 h, GFP fluorescence was observed by confocal microscope. The experiment was repeated at least three times, and a typical result is presented. **(D)** Quantification fluorescence intensity. Error bars indicate SD. a.u., arbitrary units.

HD-binding *cis*-elements (also called L1 box *cis*-elements; **Figure 7A**) can directly bind to the HD-ZIP IV class proteins (Shan et al., 2014; Xu et al., 2014; Cai et al., 2015). In the studied phenotype, *AtEDT1/HDG11* expression was modulated. The promoters of *ABI3*, *ABI5*, *EXPA5*, and *YUC6* genes were isolated and analyzed using the PLACE and Plant-CARE databases. Consistent with real-time quantitative PCR results, each of the *AtEDT1/HDG11*-overexpressing lines up-regulated genes containing HD-binding *cis*-elements in their promoters (**Figure 7B**). This result revealed that AtEDT1/HDG11 can directly or indirectly regulate these genes as a transcription activator.

To further demonstrate that AtEDT1/HDG11 acts as a transcription activator, the promoters of *ABI3*, *ABI5*, *EXPA5*, and *YUC6* were fused to an enhanced green fluorescent protein (eGFP) reporter (**Figure 7C**). These reporter constructs were co-transfected into onion cells with either the 35S-*AtEDT1/HDG11* construct as effector, or a modified *35S-AtEDT1/HDG11* with the HD domain removed, as a negative effector control. Results indicated that the promoter activity (i.e., increased GFP intensity) of *ABI3*, *ABI5*, *EXPA5*, and *YUC6* was significantly improved in the presence of the *35S-AtEDT1/HDG11* effector, but not in the negative control (**Figure 7D**). In a positive control, the GFP signal was constitutively observed in onion cells which were transfected with the CaMV35S promoter-driven construct P35S-eGFP. The result indicated that the transcription factor AtEDT1/HDG11 was able to target four genes and activated their expression *in vivo*.

## Discussion

### Improved Drought, Salinity, and Osmotic Tolerance of the *AtEDT1/HDG11*-Overexpressing Chinese Kale Was Caused by Multiple Determinants

Chinese kale is an originally Chinese vegetable belonging to the *Brassicaceae* family, and is widely cultivated in Southeast Asia; however, it is present in relatively small quantities in Europe and America (Sagwansupyakorn, 1994). It is relatively vulnerable to abiotic stresses, such as drought and high salinity, and is classified as an environmentally sensitive crop along with other leafy vegetables. In this study, we evaluated the *AtEDT1/HDG11*-overexpressing lines of Chinese kale. Our results indicated that drought and osmotic tolerance were significantly improved in transgenic Chinese kale plants. Furthermore, we investigated the factors contributed to the improved drought, salinity and osmotic tolerance in overexpressed lines.

Similar to the *AtHDG11* overexpression in *Arabidopsis* and pepper, the drought tolerance of *AtHDG11*-overexpressing Chinese kale is contributed to by multiple determinants. The *AtEDT1/HDG11*-overexpressing Chinese kale plants have a dramatically improved root system, enhanced root hair, lateral root numbers and primary root length (**Figure 1**; Supplementary Figure 3). To avoid drought-induced stress, plants improve the depth and thickness of root systems allowing them to extract water and nutrients from deep soil layers and thus to minimize the adverse abiotic stress on plant growth (de Dorlodot et al., 2007; Uga et al., 2013). The larger root system in transgenic plants should allow the enhanced root water uptake from the soil to meet the transpirational requirements of the plant (Werner et al., 2010). In addition, the water loss from transgenic plants was significantly decreased (**Figure 2E**). The majority of plant transpiration occurs through stomata and the transpiration rate can be regulated by stomatal density and/or movement (Yu et al., 2008; Kim et al., 2010). Under water deficit conditions, leaf stomatal density and guard cell size will change in response to water status (Xu and Zhou, 2008). Therefore, the lower transpiration in transgenic Chinese kale was partially attributable to a reduction in stomatal density (Masle et al., 2005; Yu et al., 2008; Yoo et al., 2010).

Likewise, stomatal aperture size is another important determinant for transpiration (Huang et al., 2009). Stomatal closure is one of the crucial ABA-regulated processes activated by dehydration conditions (Leung and Giraudat, 1998; Gonzalez-Guzman et al., 2012). Under water deficit conditions, increased cellular ABA is thought to provoke a reduction in turgor pressure of the guard cells, leading to stomatal closure and subsequent restricted transpiration as a mechanism to adapt to water deficiency (Zhu, 2002). Compared to the corresponding wild-type plants, the transgenic lines guard cells were more sensitive to ABA and drought stress, which led to quick stomatal closure. The smaller stomatal aperture may contribute to reduced water loss from the plant cells, thus enhancing osmotic stress tolerances in the transgenic plants (Huang et al., 2009). The observation that *HDG11* up-regulation leads to the ABA hypersensitivity phenotype has not been reported previously until this study; such observation can help us better understand the mechanism of reduced water loss in the transformed lines compared to the untransformed lines.

Drought and osmotic stress can cause oxidative and osmotic damage in plants (Zhu, 2002; Carvalho, 2008). The transgenic plants were better protected from osmotic and oxidative damage by increasing proline and SOD (**Figures 2H,I and 3E,F**). ROS was overproduced under various environmental stressors such as drought and osmotic. The significantly reduced H_2_O_2_ levels detected in the transgenic Chinese kale, under both drought and osmotic conditions (**Figures 2J and 3G**), indicate that they are more efficient in oxidative scavenging, which contributed to reduced oxidative damage in the transgenic plants.

### Molecular Mechanisms Underlying the Drought Tolerance and Auxin Overproduction Phenotypes of *AtEDT1/HDG11*-Overexpressing Lines

The HD-ZIP transcription factor, AtHDG11, was first found to be involved in trichome branching and other aspects of development (Nakamura et al., 2006; Khosla et al., 2014). Homeodomain transcription factor can directly target HD-binding *cis*-elements (also called L1 box *cis*-elements), which has been widely documented (Shan et al., 2014). Transcriptomes were compared between the wild-type and *edt1D Arabidopsis* roots, and the results revealed that several gene families of cell-wall-loosening proteins and jasmonate biosynthesis and signaling pathways were upregulated in the *edt1D* root (Xu et al., 2014; Cai et al., 2015). Most of these genes contain HD-binding *cis*-elements in their promoters predominantly with the TTTAATTT sequence, which can be bound by HDG11 *in vitro* and *in vivo*. Similar to *Arabidopsis edt1D* we also detected *EXPANSIN A5* (*EXPA5*) up-regulated in *AtHDG11* overexpression lines. This may contribute, in part, to improved primary root elongation in transformed Chines kale plants. Similarly, in the cotton HD-ZIP, the transcription factor GhHOX3 controls cotton fiber elongation directly by regulating wall loosening protein genes GhRDL1 and GhEXPA1 (Shan et al., 2014). This indicates homeodomain transcription factor has a conserved function in regulation, regulating downstream genes via directly targeting the L1-box *cis*-element.

The *AtEDT1/HDG11*-overexpressing lines improvement of drought and osmotic stress was partly attributable to the regulation of a set of stress-related genes. In this study, we found *AtEDT1/HDG11* significantly up-regulated the stress response of related genes and transcription factors containing at least one L1-box *cis*-element (data not shown). We found that ABA synthesis pathways (*NCED3* and *LOS5/ABA3*), the Proline synthesis gene *P5CS* and stress tolerance-related genes *RD29A*, *LEA*, and Cu/Zn *SOD* were significantly upregulated in transgenic plants under both normal and drought stress conditions (**Figure 6B**), similarly to previous studies on *Arabidopsis* and rice (Yu et al., 2008, 2013). In addition, the expression levels of six stress related transcription factors *ABI3*, *ABI5*, *DREB2*, *SNAC1*, *NAC3*, and *NAC5* (Liu et al., 1998; Finkelstein and Lynch, 2000; Lopez-Molina et al., 2002; Hu et al., 2006), were also found to be significantly up-regulated in *AtEDT1/HDG11*-overexpressing plants (**Figure 6B**). It is known that ABI3, a B3-domain transcription factor and ABI5, a basic leucine zipper transcription factor, have been assigned roles in ABA signaling largely based on their involvement in late seed development, particularly in the ABA-dependent induction of LEA genes in the final stages of seed development (Finkelstein and Lynch, 2000; Battaglia et al., 2008). Studies in transgenic plants indicate overexpression of the transcriptional regulators ABI3 or ABI5 that confer hypersensitivity to ABA (Himmelbach et al., 2003; Zou et al., 2008). Moreover, since ABI3 and 14–3–3 proteins are able to form complexes with the bZIP protein ABI5, the resulting dimers interact to regulate the expression of ABA-controlled genes via binding to ABA responsive elements (ABREs) located in their promoters (Finkelstein et al., 2002). It is possible that up-regulation of *ABI3* and *ABI5* in transgenic Chinese kale augments the ability to respond to stress signals and regulates ABA-induced gene expression (Yu et al., 2008). Interestingly, digital gene expression profile (DGE) analysis of *ABI3* and *ABI5* genes in the ZH11 wild type and *AtEDT1/HDG11*-overexpressing rice did not detect significant results, potentially because these are different species.

We found that the auxin biosynthesis *YUC* gene family and auxin transport *PIN* gene family were upregulated in *AtEDT1/HDG11*-overexpressing Chinese kale lines, though not reported in *Arabidopsis* and rice (Yu et al., 2008, 2013; Xu et al., 2014; Cai et al., 2015). The YUC gene family encodes flavin monooxygenase enzymes which catalyze a rate-limiting step in auxin biosynthesis. It is known that auxin has profound effects on plant growth and development (Zhao, 2010; Kim et al., 2013). Overexpression of the *YUC* family gene in *Arabidopsis* and other species always leads to similar auxin overproduction phenotypes, which is consistent with observations in the present study (**Figure 1**; Supplementary Figure 2). Similar to the auxin biosynthesis gene, we found transport gene PINs were also upregulated in *AtEDT1/HDG11*-overexpressing lines. It widely known that PINs plays an important role in controlling auxin polar transport and regulating cell division and cell expansion in the primary root (Blilou et al., 2005; Wiśniewska et al., 2006). This may be attributed, to an extent, to the formation of a larger root system in transformed lines. In addition to regulating development, auxin biosynthesis, and transport genes were also involved in stress tolerance. Many auxin biosynthesis, transporter and response genes are involved in plant responses to biotic and abiotic stresses, including drought, high salinity, and pathogen infection (Woo et al., 2007; Ye et al., 2009; Blomster et al., 2011; Cheol Park et al., 2013). Overexpression of *AtYUC6* and *AtYUC7* in *Arabidopsis* result in auxin-overproduction phenotypes and up-regulation of stress-response genes, significantly elevating tolerance to drought stress (Lee et al., 2012; Ke et al., 2015). Similarly, the ectopic expression of *AtYUC6* in potato also involved auxin overproduction phenotypes and enhanced drought tolerance (Kim et al., 2013). On the contrary, the T-DNA insertional rice mutants in a *CONSTITUTIVELY WILTED1* (*COW1*) gene, which encodes a new member of the YUC protein family, exhibit water-deficient phenotypes of rolled leaves and reduced leaf widths that could result from lower root to shoot ratios which ultimately lead to insufficient water uptake (Woo et al., 2007). Recently, the *Arabidopsis* plants overexpressing *YUC6* displayed enhanced IAA-related phenotypes and exhibited improved drought stress tolerance, low rates of water loss and controlled ROS accumulation under drought and oxidative stresses (Cha et al., 2015). This result demonstrated a double function of YUC6, which acts as a flavin monooxygenase in auxin biosynthesis and as a FAD- and NADPH-dependent thiol-reductase in the stress response. These studies demonstrate that the stress-related phenotype observed in plants that overexpress YUC6 is not based on IAA overproduction but on its activity as a thiol-reductase. In this study, the regulation of YUC6 and its family members by HDG11 suggests that up-regulation of YUCs in the HDG11 *Brassica* overexpression lines may not only control auxin biosynthesis but also enhance ROS scavenging under drought and osmotic stress conditions (**Figures 2J** and **3G**).

Taken together, our study indicated that overexpressed *AtEDT1/HDG11* in the Chinese kale enhances drought, salinity, and osmotic tolerance and improves biomass. We have analyzed expression patterns of several important auxins, ABA, and stress-related genes. Future transcriptome analysis of the wild-type and the transgenic Chinese kale should help to uncover other key genes involved in abiotic tolerance and plant development.

## Author Contributions

Conceived and designed the experiments: ZZ, JL, and CC. Performed the experiments: ZZ, XX, and BS. Analyzed the data: HC, ZZ, and LZ. Wrote the paper: ZZ, JL, and CC. Revised the manuscript: CC and BC.

## Conflict of Interest Statement

The authors declare that the research was conducted in the absence of any commercial or financial relationships that could be construed as a potential conflict of interest.

## Footnotes

1 www.dna.affrc.go.jp/PLACE/

2 http://bioinformatics.psb.ugent.be/webtools/plantcare/html/
