# Supplementary material for: Overexpression of AtEDT1/HDG11 in Chinese Kale (Brassica oleracea var. alboglabra) Enhances Drought and Osmotic Stress Tolerance
Source: Front Plant Sci. 2016 Aug 30;7:1285. doi: 10.3389/fpls.2016.01285 (PMC5003845; doi:10.3389/fpls.2016.01285)
Supplement: Supplementary file 1 [file Data_Sheet_1.DOCX]

Supplementary Material

**Overexpression of *AtEDT1/HDG11* in Chinese kale (*Brassica oleracea* var. *alboglabra*) enhances drought and osmotic tolerance**

Zhangsheng Zhu^123^, Binmei Sun^13^, Xiaoxia Xu^123^, Hao Chen^123^, Lifang Zou^123^, Guoju Chen^123^, Bihao Cao^123^, Changming Chen^123*^ and Jianjun Lei^123*^

**^*^**1 College of Horticulture, South China Agricultural University, Guangzhou, China

2 National Engineering Research Center of Plant Space Breeding, SCAU, Guangzhou, China

3 Key laboratory of Biology, Innovation and Utilization for Germplasm Resources in Horticultural Crops in Southern China, Guangzhou, China

**^*^** Corresponding authors: Email: jjlei@scau.edu.cn, cmchen@scau.edu.cn，Tel: 86-2085288275. Fax: 86-2085280228.

## Supplementary Figures


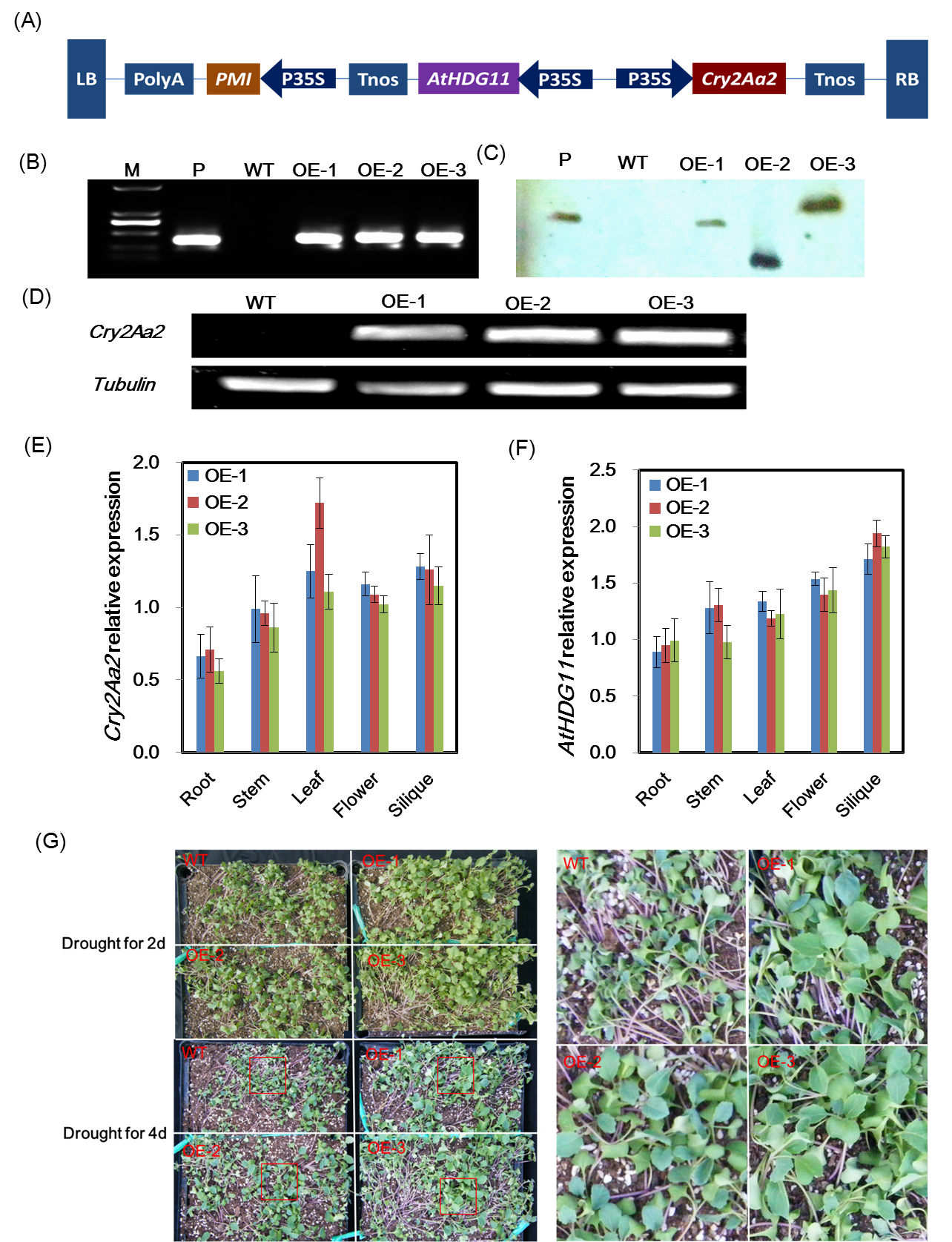


**Supplementary Fig. 1** Generation of overexpressing Chinese kale plants. Transgenic Chinese kale plants were generated by *Agrobacterium*-mediated transformation using the Chinese kale inbred line 25 as the recipient. To identity transgenic plants, gnomic DNA was isolated from the putative transgenic and wild type Chinese kale. The PCR was used to screen for putative transgenic plants with the primers Cry2Aa2-FOR and Cry2Aa2-REV (Supplemental table 1). RT-PCR was performed to analysis *Cry2Aa2* (AF433645.1) expression pattern. To further analyze the expression levels of *AtEDT1/HDG11* and *Cry2Aa2* in transgenic plants, total RNA was extracted from both transgenic and wild type seedlings using TRIzol reagent (Life, USA). One μg of RNA from each sample was used for the reverse transcription reaction by PrimeScript^TM^ RT reagent kit with gDNA eraser (Takara, Japan). The qRT-PCR was performed on a LightCycler 480 qRT-PCR system (Roche, Switzerland). The program was 94 °C for 8 min, followed by 45 cycles of 94 °C for 20 s, 56 °C for 20 s, and 72 °C for 30 s. The Chinese kale *Tubulin8* expression level was used as an internal control.

12 T_0_ independent transgenic plants were generated, southern bloting analysis indicated most transgenic lines harboring one copy. Positive T_2_ homozygous transformants were prescreened for drought resistance in the greenhouse and more than 80% transgenic lines showed improved drought tolerance during seedling stage. Several drought tolerance lines (i.e. OE-1, OE-2, OE-3) were selected for further analysis. RT-PCR analysis indicated that *Cry2Aa2* was expressed in all of the three lines, whereas no signals were detected in wild-type plants. (A) T-DNA region of expression vector PCAMBIA1301-Cry2Aa2-AtHDG11-PMI. LB left border, RB right border, *PMI* phosphomannose isomerase gene, P35S Cauliflower mosaic virus (CaMV) 35S promoter, polyA (CaMV) 35S polyA, Tnos 3-termination signal of nopaline synthase. (B) PCR confirmed of T_0_ putative transgenic plants: Lane 1, 2 kb marker; lane 2, pCAMBIA1301-Cry2Aa2-HDG11-PIM plasmid; lane 3, wild-type (WT); lanes 4–6, transgenic plants. (C) Southern bloting analysis indicate transgenic lines OE-1, OE-2 and OE-3 harboring one copy. P positive control, WT wild-type plants, OE-1, OE-2 and OE-3 transgenic plants. (D) RT-PCR analysis of *Cry2Aa2* expression levels in T_0_ transgenic plants, whereas no signals were detected in wild type plant. (E) and (F) qRT-PCR analysis of *Cry2Aa2* and *AtEDT1*/*HDG11* expression in T_2_ transgenic plants root, stem, leaf, flower, and silique, respectively. The relative transcript level was obtained as folds of the *Tubulin8* transcript levels as the internal control. Values are mean ± SD (n =3).


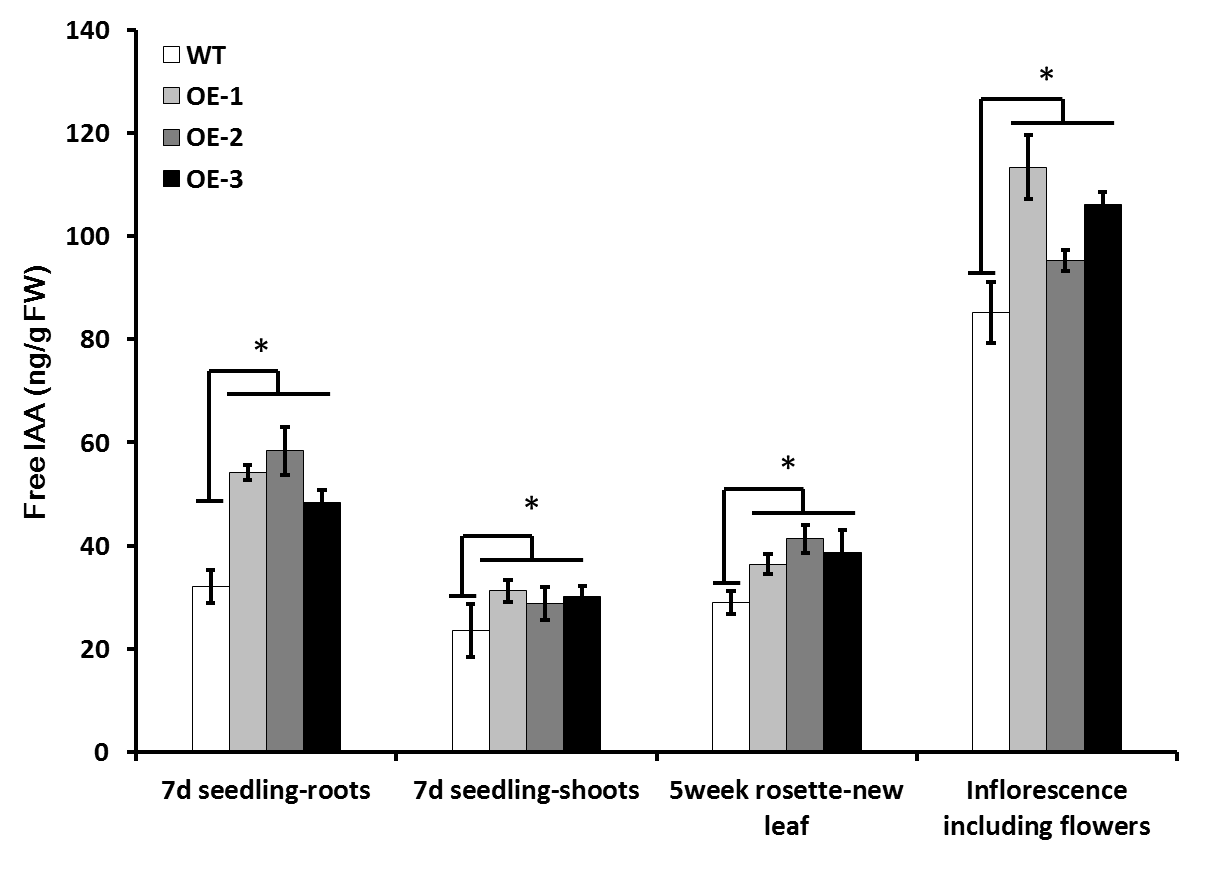


**Supplementary Fig. 2** Free IAA measurement in different organs of *AtHDG11*-overexpressing lines and wild type. Specified organ tissues from wild type and AtHDG11-overexpressingwere harvested and used for free IAA measurement. Values are mean ± SD for three replicates. ** P*< 0.05 asterisks indicate Student’s *t*-test significant differences.


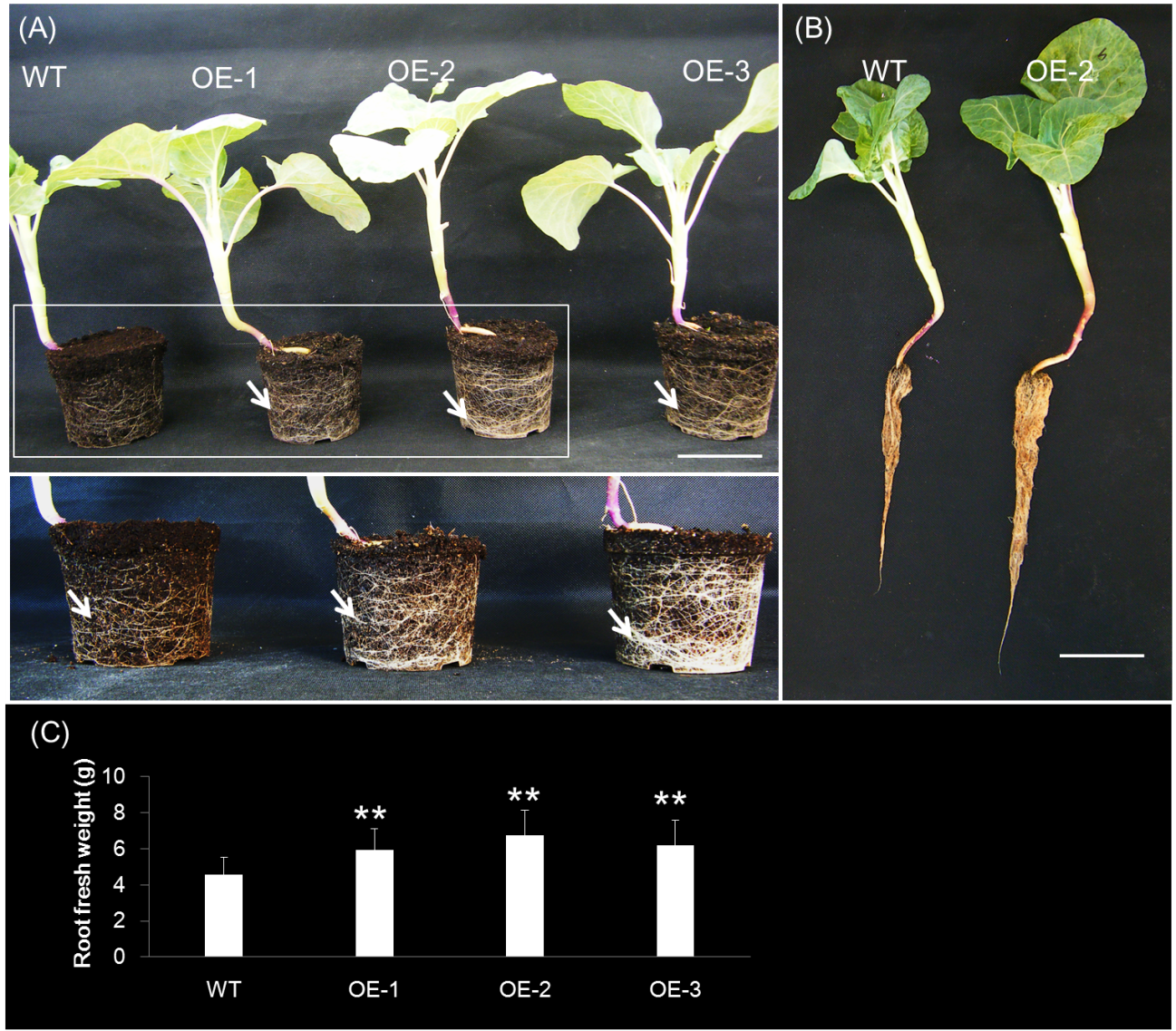


**Supplementary Fig. 3** Root morphology of 8-week-old transgenic and wild-type Chinese kale plants. Transgenic and wild-type plants root without (A) or with (B) soil detached. Bars = 8 cm. (C) Fresh weight of 8-week-old transgenic and wild-type plants. Values are mean ± SD (n= 10, *** P* < 0.01). Asterisks indicate Student’s *t*-test significant differences.


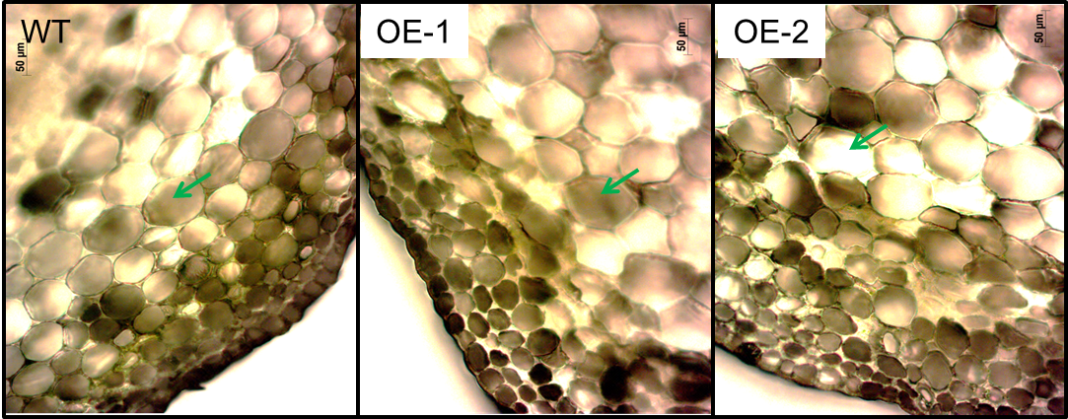


**Supplementary Fig. 4** Stem cross section of transgenic and wild type Chinese kale plants. The cells in stem of transgenic plants is larger than wild-type plants. Bar= 50μm.


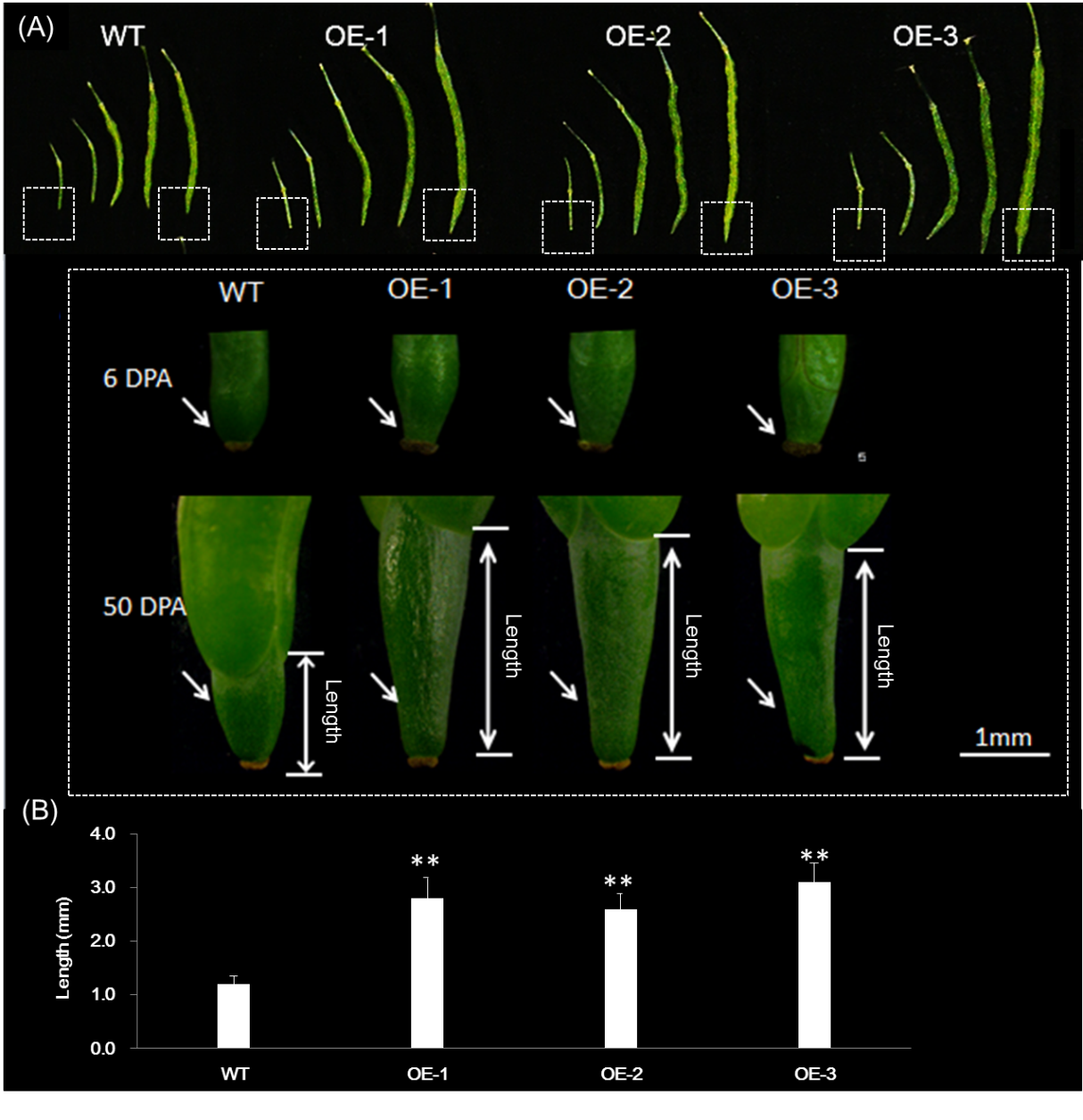


**Supplementary Fig. 5** Transgenic plants exhibit siliques with altered tip morphology. (A) 6 DPA and 55 DPA (day post anthesis, DPA) siliques of wild-type and transgenic Chinese kale plants. (B) Apex length of 55 DPA siliques as shown in (A). Values are mean ± SD (n= 50 siliques, *** P* < 0.01). Asterisks indicate Student’s *t*-test significant differences.


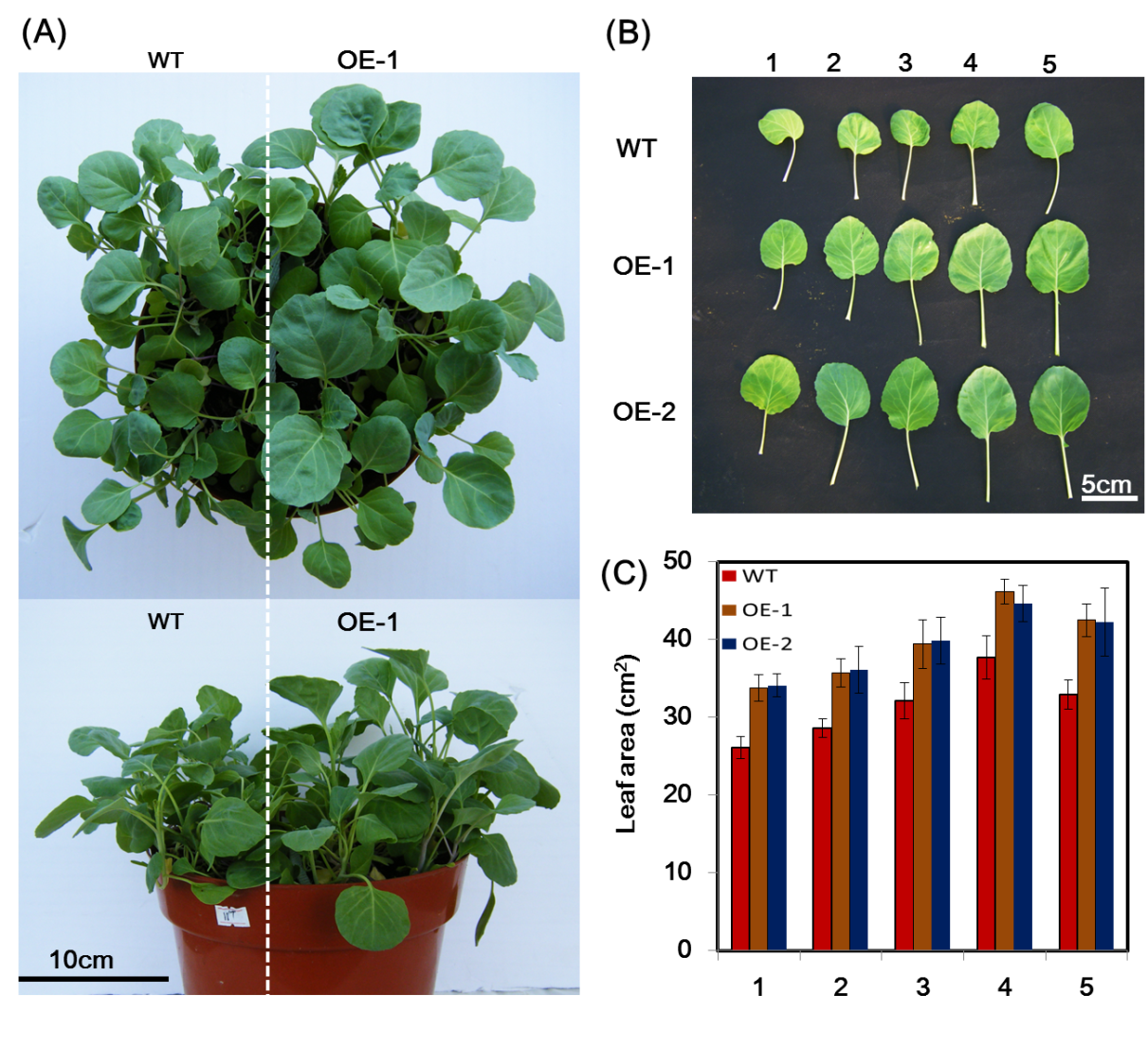


**Supplementary Fig. 6** Growth morphology of Chinese kale plants during seedling stage. (A) Transgenic and wild type Chinese kale in seedling stage. Bar= 10cm. (B) The 35 d old transgenic and wild type plants of first to fifth leaf. Bar= 5cm. (C) The first to fifth leaf area of transgenic and wild-type plants as shown in (B). Values are mean ± SD (n= 10).


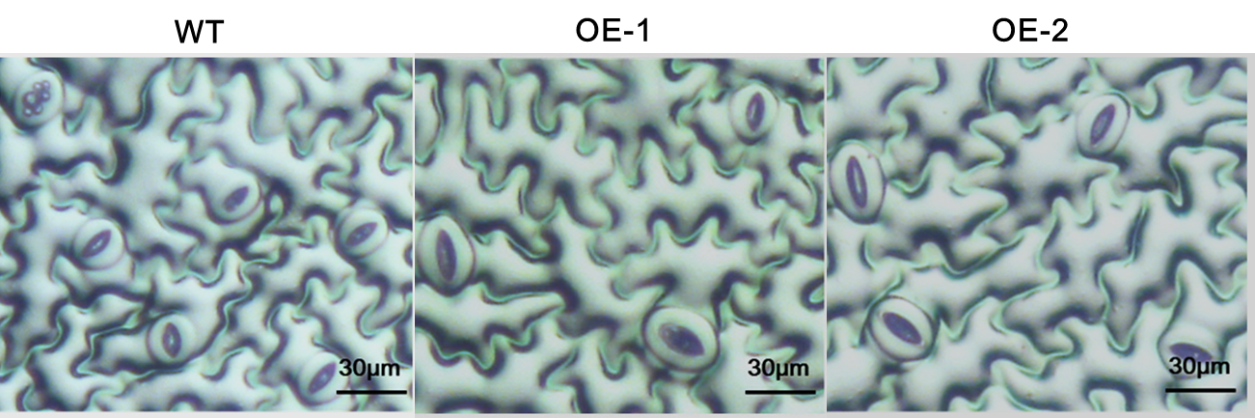


**Supplementary Fig. 7** Comparisons of abaxial epidermal imprint images of the wild-type and transgenic Chinese kale at the ×100 magnifications. Scale bar = 30μm.

## Supplementary table

**Supplement table 1** Primers used in this paper

| **Name** | **Sequence ( 5’ to 3’)** | **Accession numbers** |
| --- | --- | --- |
| *AtHDG11-FOR* | **GCTCTAGA**ATGAGTTTCGTCGTCGG | NM_105996 |
| *AtHDG11-REV* | **TCCCCCGGGT**CAAGCTGTAGTTGAAG |  |
| *Cry2Aa2-FOR* | GGAACAGGGTTTTGAGTAG | FJ788388.1 |
| *Cry2Aa2-REV* | GATATTGAGTGAATTATGGG |  |
| *Tubulin8-F* | CTTCTTTCGTGCTCATTTTGCC | XM_013743003 |
| *Tubulin8-R* | CCATTCCCTCGTCTCCACTTCT |  |
| *qABA3-R* | GATAAATGGTCCTGAACTTGC | LOC106295608 |
| *qABA3-F* | ATCTCCTGAAGACTACAGTTGC |  |
| *qABI3-F* | ACGATGGCGTTGAGGGGAGC | XM_013771836 |
| *qABI3-R* | CGGAGGGTTGTGGAATACTG |  |
| *qABI5-F* | AGTTCCTCGTCTCTATTTG | JX870620.1 |
| *qABI5-R* | CTATCCCTGGCTTCTTATT |  |
| *qERECTA-F* | CTCGAGCAGCACTATCCCGT | XP_013605100 |
| *qERECTA-R* | GCCTATTCCCTCTCTCCCTT |  |
| *qEXPA5-F* | TGCCACTAACTTTTGTCCTCCT | NM_001301916 |
| *qEXPA5-R* | GTAAACCTTATCCCGCCTCTTC |  |
| *qNCED3-F* | TCAGATTCTTTGGTTTTTGGG | XM_013732216 |
| *qNCED3-R* | TCCGCCTTGAGTTATTCTTGC |  |
| *qP5CS-F* | TGAGTTAGGGTCACTTGGAGG | XM_013781065 |
| *qP5CS-R* | GAGGAGGTTTTGGTAGGCGGG |  |
| *qSOD-F* | TTCTACTTACCACAAGCAACACG | DQ431853 |
| *qSOD-R* | ACCTTCACAATCACTGACAGCCA |  |
| *qDREB2-F* | AGCCAGATGCGGCGTTGGTGTG | XM_013762554 |
| *qDREB2-R* | AGCGTGCGGGACGGATAGTGTT |  |
| *qCry2Aa2-F* | GGAACAGGGTTTTGAGTAGGGT | FJ788388.1 |
| *qCry2Aa2-R* | GATATTGAGTGAATTATGGGGGA |  |
| *qRD29A-F* | CACACTCCCGCCTCTCTCCTC | XM_013770906 |
| *qRD29A-R* | AACAATCGCCGGTACTCCAGC |  |
| *qLEA-F* | GGCGTCAAAGCAACAAAGCC | DQ178983 |
| *qLEA-R* | GATAACCCTCGTGCCCCAGC |  |
| *qSNAC1-F* | AATGGGGAGAGCTCGAAGAT | AY245879.1 |
| *qSNAC1-R* | CCTGGAAGGGAGTGAAATAA |  |
| *qNAC3-F* | CCCAACTAAGTTTACCAC | AB049070.1 |
| *qNAC3-R* | AATACCATTCTTTTTCCC |  |
| *qNAC5-F* | ATATGATCCTTGGGACCTTC | JF957837 |
| *qNAC5-R* | GCCTTCTTTATACCGACTGG |  |
| *qHDG11-F* | GGTTCAGGGCTCTTATG | NM_105996 |
| *qHDG11-R* | GGAACAGGGTTTTGAGTAG |  |
|  |  |  |
| *qYUC3-F* | AAATACGGTCTAAAAAG | XM_013748010 |
| *qYUC3-R* | AGAATAACGGAATCAAT |  |
| *qYUC5-F* | GGTGTTCCCTTTGTGGTA | XM_013741164 |
| *qYUC5-R* | GGATAATCGGGGTAGTTT |  |
| *qYUC6-F* | CCTTTCCCGACCACTTCC | XM_013844550 |
| *qYUC6-R* | TTCACCCACGCTCACCAC |  |
| *qYUC7-F* | CCAATCTACCCTTCCCCG | XM_009145633 |
| *qYUC7-R* | CTCCACAGCCCAAACCTC |  |
| *qYUC8-F* | CAAAAGGTTTAACGGAA | XM_013782915.1 |
| *qYUC8-R* | GTAGCCAATAAGGGACG |  |
| *qPIN1-F* | GGGGAATAGTAACGATA | XM_013760601 |
| *qPIN1-R* | TCCACTTGAAGGAGATG |  |
| *qPIN2-F* | GAGATGTTTACTCGCTTC | XM_013763840 |
| *qPIN2-R* | TTCACCACTAATGTTTTT |  |
| *qPIN3-F* | CACGCTTCTCCTTTTCCT | XM_013846397 |
| *qPIN3-R* | TTCCCGTCGTCTCCTATC |  |
| *qPIN4-F* | GCACTGCGGTTATTTTTG | XM_013765718 |
| *qPIN4-R* | CCTTTGTTTATTTCCTGG |  |
| *qPIN7-F* | TCTTTTTGCGGTGCTAAC | XM_013743929 |
| *qPIN7-R* | AACCATCCCACAACTCTC |  |
